# Digital Health Convergence Workshop: The Kenyan experience

**DOI:** 10.1093/oodh/oqae014

**Published:** 2024-04-09

**Authors:** Emma Waiyaiya, Job Nyangena, Ryan Nyotu, Joyce Wamicwe, Nelly Nyaga, Steven Wanyee, Bernard Langat

**Affiliations:** Digital Health Innovation, Kenya Health Informatics Association (KeHIA), One Padmore Place, George Padmore Road, P.O. Box 40664, 00100 Nairobi, Kenya; Digital Health, Informatics, Policy, and Research, Ministry of Health, Afya House, Cathedral Road, P.O. Box 30016, 00100 Nairobi, Kenya; Digital Health, Informatics, Policy, and Research, Ministry of Health, Afya House, Cathedral Road, P.O. Box 30016, 00100 Nairobi, Kenya; Digital Health, Informatics, Policy, and Research, Ministry of Health, Afya House, Cathedral Road, P.O. Box 30016, 00100 Nairobi, Kenya; Data Science, Research and Innovation, IntelliSOFT Consulting, One Padmore Place, George Padmore Road, P.O. Box 41664, 00100 Nairobi, Kenya; Executive Committee, Kenya Health Informatics Association (KeHIA), Nairobi, Kenya; Digital Health, Informatics, Policy, and Research, Ministry of Health, Afya House, Cathedral Road, P.O. Box 30016, 00100 Nairobi, Kenya

**Keywords:** health information exchange, enterprise architecture, universal health coverage, Convergence Workshop, standards and interoperability, digital public infrastructure for health

## Abstract

In the dynamic landscape of digital development, the power of collaboration and convergence emerge as significant forces driving innovation and excellence. These forces not only align with global best practices defined in the Principles for Digital Development but also propel advancements in healthcare. A Convergence Workshop, which brings together diverse stakeholders, is an approach to kick-start and sustain a shared vision within the ecosystem—from healthcare providers to government agencies—and serves as a catalyst for consensus building on critical aspects, including data standards, security protocols and regulatory compliance. The value of Convergence Workshops lies in their ability to foster collaboration, address multifaceted challenges and pave the way for a unified, interoperable health information exchange and overarching digital public infrastructure for health while building relationships and trust between stakeholders. In this context, they play an indispensable role in shaping the future of a country’s healthcare ecosystem, enhancing data-driven decision-making and ultimately improving health outcomes.

## INTRODUCTION

African countries have realized the potential for digital health as a potent tool to tackle the health system challenges they face, and as such, they are investing in ambitious national digital health transformation efforts [1]. Digital health technologies and data in health care have been shown to enable and catalyse the achievement of universal health coverage (UHC), a cornerstone goal for governments aiming to ensure the health and well-being of their populations [2, 3]. However, as national governments embark on their healthcare digitization journey and lay the groundwork for digital infrastructure, they are confronted with ‘e-chaos’, characterized by (i) significant fragmentation and poorly coordinated implementation, (ii) a lack of clear frameworks for standards and interoperability and (iii) structural misalignment caused by a mismatch of country priorities and investments available [4, 5]. These challenges can sometimes feel daunting for governments keen to drive change.

Yet, a compelling case can be made, both in terms of economic and health outcomes, for investing in a structured and standardized health information exchange (HIE) built upon robust standards and interoperability principles [6]. Specifically, the availability of accurate and timely information at the point of contact between the health system and a patient leads to reduced cost, better-quality care and increased patient experience [7]. A functioning HIE is an example of digital public infrastructure for health (DPI-H) where DPI is defined as ‘solutions and systems that enable essential society-wide functions and services’, with DPI-H described as the ‘health-specific components of a country’s digital infrastructure that enable an ecosystem of inclusive, scaled, user-driven digital applications in a health system’ [8].

‘A journey of a thousand steps begins with a single step’, and we posit that the most meaningful first step in this crucial journey towards establishing a national HIE is a convergence of ideas, capacities, resources and all stakeholders—governments, private sector, academia, investors and implementers—to be facilitated in an open and enabling environment to learn from each other and, together, co-create, define and commit to a set of priorities to move a country forward in delivering on its shared vision for healthcare digitization.

## THE JOURNEY TOWARDS A NATIONAL HIE

Countries’ digital health maturity levels vary, resulting in a need for contextual and tailored approaches to planning and executing HIEs. As governments embark on this journey, they can learn from their past policy decisions, previous implementation methods and experiences gained [7, 9, 10]. Notably, African countries such as Kenya, Tanzania, Rwanda and Malawi are on this journey [11–13].

The aim of this article is not to evaluate any country’s chosen path towards a foundational architecture for HIE or DPI - H, but rather to highlight and emphasize how strategic and crucial a Convergence Workshop is, as one of the first important steps in harmonizing priorities and co-creating a shared vision and a clear roadmap towards establishing an enterprise architecture.

## A CASE EXAMPLE FOR KENYA

The ‘Digital Superhighway’ is a bold and ambitious Kenyan initiative to create a robust national DPI across Kenya, specifically focusing on four key pillars: health; agriculture; micro-, small- and medium-sized enterprises and financing [14, 15].

In alignment with the global commitment to UHC and the recognition of digital transformation as a key enabler, the Government of Kenya enacted the Health Act 2017 and the Digital Health Act 2023, which mandated the establishment and maintenance of a Comprehensive Integrated Health Management Information System (CIHMIS). At the core of this integrated health information system is a national HIE, as envisioned in the Digital Health Superhighway, that enables the secure and seamless flow of electronic health information across the healthcare system. This national HIE is a step towards promoting patient-centred healthcare accessible to all citizens, in alignment with the government’s vision to revolutionize public service through digitization.

In 2013, Kenya transitioned into a devolved system of governance comprising two levels: the national government and 47 semi-autonomous county governments. Under devolution, the health service delivery function was transferred to county governments, while the national government retained policy and regulatory functions [16, 17]. A key aspect of establishing the Kenyan Digital Health Superhighway is the articulation of clear roles within the existing public health administrative system.

It is crucial to clarify that the Kenyan Ministry of Health’s (MOH) primary responsibility is to provide the HIE, oversee the interoperability layer and establish standards for the development, implementation and maintenance of the CIHMIS. Meanwhile, the 47 devolved counties would be responsible for the digitalization of the health facilities under their mandate according to the standards established nationally. By defining these distinct roles for both the national government and the county administrations, the MOH aims to enhance coordination and facilitate effective resource mobilization. Ultimately, this approach fosters greater ownership and commitment from all 47 devolved county governments. In addition, from the very outset, the design and implementation of CIHMIS prioritize considerations of adoption and change management, strengthening the rationale for the convergence approach.

To this end, the Kenyan MOH, through its Directorate of Digital Health, Informatics, Policy and Research (DHIPR), convened a 5-day Convergence Workshop in July 2023. This case example will detail the approach taken to design, implement and conclude the workshop, as well as highlight the lessons learned and outcomes of the workshop.

## METHODOLOGY OF A CONVERGENCE WORKSHOP

A Convergence Workshop is a process of converging people, resources, tools and technologies available for each country to help strengthen a digital health vision. All stakeholders are gathered in an open environment where participants from different backgrounds come together to learn more about new concepts, tools or solutions available in healthcare to achieve quality of care with digital health.

According to the ‘Digital Health Convergence Meeting Toolkit—2018’ [18], there are six recommended steps, adaptable based on context, for designing a digital health Convergence Workshop: (i) request, (ii) coordination, (iii) planning, (iv) preparation, (v) implementation and (vi) follow-up.

### Request

The MOH, through its DHIPR, formally requested technical and financial support from local digital health champion institutions and its development partners, such as the Kenya Health Informatics Association, University of Nairobi’s HealthIT project, United States Agency for International Development, Tony Blair Institute for Global Change and the Pan African Health Informatics Association (HELINA), among others, to plan and execute the Convergence Workshop. The core technical group met to align with the government’s vision, objectives and expectations of the Digital Health Superhighway.

### Coordination

The core planning and strategy team comprised a lean group of individuals, with leadership and coordination provided by the DHIPR. This team was responsible for directly coordinating with partners and stakeholders on any support required, organizing the workshop, running it and generating a workshop report that would be used to guide the implementation of the Digital Health Superhighway.

### Planning

The priority goal was determined to be the establishment of a national HIE. Tools such as the Global Digital Health Monitor, World Health Organization (WHO) Digital Health Atlas, and the Mind the GAPS framework were identified as essential for curating and refining the Kenyan situational analysis, fostering alignment among all stakeholders [19–21]. Two recommended steps in the planning phase are (i) defining stakeholders and (ii) stakeholder engagement.

The core group set out, through iterative consultation, to define the list of internal stakeholders—those within the MOH, the Ministry of Information and Communications Technology and the Digital Economy (MOICT), the Council of Governors and other government parastatals, such as the Ministry of Interior, academia, and technical working groups, and the list of external stakeholders—development and implementing partners, UN organizations and special population representatives.

The stakeholders were identified based on their understanding, knowledge and experience of the HIE and could offer diverse perspectives in digital health. Stakeholders were engaged through a series of consultative and collaborative virtual meetings, and the core group was empowered to designate roles for the various multidisciplinary groups, as shown in Table 1.

**Table 1:** Examples of stakeholder categories and various roles designated to stakeholders

Stakeholder	Roles
Policymakers (e.g. MOH)	• Set the overall direction and objectives for the HIS • Ensure alignment with the country’s health plan • Provide political commitment to the HIS • Ensure that required resources are accessible • Coordinate with other ministries involved in the process
Directors (health information system, health management information system, eHealth unit, health systems strengthening unit) (e.g. technical working group heads, academia)	• Provide support to the implementation group • Ensure that the progress is aligned with the planned activities • Collaborate with other stakeholders involved • Report progress to policymakers
Ministry of Information and Communications Technology	• Provide background information on the setting and capabilities of current programs • Provide necessary input to the proposed programs • Ensure that proposed programs are within reach
Legal (e.g. legal advisors)	• Provide legal input and ensure legal compliance with the planned activities • Provide the necessary guidance
Program managers (e.g. implementing partner organizations)	• Identify the requirements of the proposed programs
Development partners (e.g. donor partners and UN organizations)	• Support the requirements
Special population representatives (e.g. representatives of vulnerable, marginalized and excluded populations)	• Provide diverse perspectives and insights on issues relevant to their communities

Source: Digital health convergence meeting toolkit – 2018 [18], adapted by localization of stakeholders and inclusion of special population representatives

### Preparation

The preparatory phase began with (i) the articulation and recognition of the digital health vision and (ii) the development of an agenda for the workshop. The Kenyan MOH’s vision is to implement a Digital Health Superhighway with a standardized HIE [22]. Under this guidance and direction, the objectives and expected outcomes for the Convergence Workshop were established.

The specific objectives for the Convergence Workshop were (i) to outline the government’s vision for the digitalization of healthcare in Kenya; (ii) to align the health sector’s digital health priorities, roles and responsibilities around the Digital Health Superhighway and (iii) to develop a roadmap for the implementation of Kenya’s HIE.

A 5-day agenda was developed, including keynote speeches, panel discussions, formal presentations, breakout and feedback sessions, action setting and prioritization sessions, all geared towards achieving the set objectives and ensuring participants remain engaged.

### Implementation

#### Convene workshop

The digital health Convergence Workshop was convened on 17–21 July 2023.

**Figure 1:**
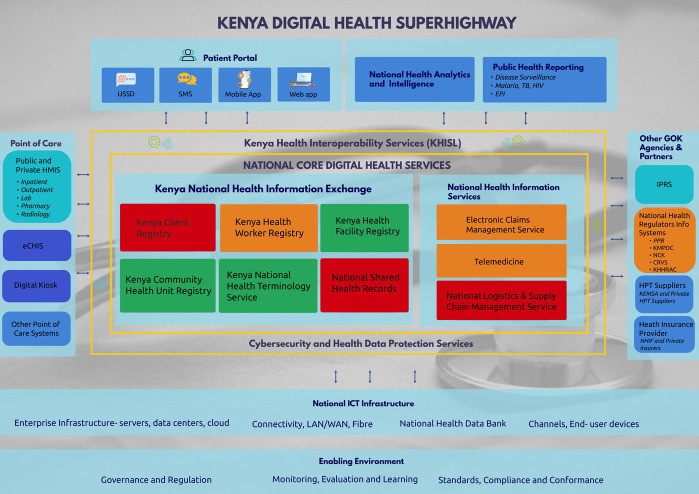
An illustrative view of the vision of Kenya's national Health Information Exchange, also known as the ‘Kenyan Digital Health Superhighway’

An illustrative view of the vision of Kenya's national HIE was shared as shown in Fig. 1.

On the first day of the workshop, the DHIPR team articulated the Ministry’s digital health vision and its alignment with the government’s digital transformation economy priority [22, 23]. The partners and stakeholders interrogated and engaged the MOH officials to get clarity on the vision and shared preliminary insights on the true state of digital health in Kenya presently. Panel discussions and presentations were held to assess the status of the enabling environment and the maturity of Kenya’s digital health context.

There were 2 days of focused group discussions where all participants self-selected into one of 12 active digital health work streams, defined on the core areas for discussion. Each work stream was facilitated by domain experts and members who expressed interest in the core topic. The work streams, as listed in Table 2, included registries such as Health Worker Registry, Client Registry, Facility Registry and Community Health Unit Registry; Components such as Shared Health Record, Health Financing Services, Logistics Management and Product Catalogue, Terminology Services, Point of Care systems such as Health Management Information System; Emerging technologies areas such as Artificial Intelligence and Cybersecurity; Key enablers such as Standards and Interoperability, Policy, Legislation and Governance and Infrastructure.

**Table 2:** A list of the 12 work streams developed for the Kenyan Convergence Workshop

Registries	1) Client Registries 2) Health Worker Registries 3) Facility Registries and Community Health Unit Registries
Components	4) Shared Health Record 5) Health Financing Services 6) Logistics Management, Product Catalogue and Terminology Service 7) Point of Care systems such as Health Management Information Systems
Emerging Technology Areas	8) Artificial Intelligence 9) Cybersecurity
Key Enablers	10) Standards and Interoperability 11) Legislation, Policy, Compliance 12) Infrastructure

**Table 3:** The priority activities and outcomes as defined at the conclusion of the Convergence Workshop held in Kenya in July 2023

**Kenya Convergence Workshop,** **Priorities July 2023**
Leadership & Governance • As part of the governance framework of the Digital Health Superhighway, a reorganization and realignment of the initial ‘e health Technical Working Groups’ is underway to (1) clearly define their scope of work in alignment with the 12 digital health work streams identified in the Convergence Workshop, (2) encompass a wider array of stakeholders, notably incorporating the COG, responsible for coordinating activities across the 47 devolved counties and (3) rename the existing ‘eHealth Technical Working Groups’ to the ‘Digital Health Technical Working Groups’ to better reflect their enhanced role and significance. • Technical Working Groups to have open membership of stakeholders in digital health with a clear schedule of meetings and co-chaired by the MOH and COG. Strategy & Investments • Finalization of an MOH-led, costed National Digital Health Superhighway with clear timelines, roles & responsibilities (short, medium and long term) • Buy in unlocked from key donors with funding aligned to a wholistic digital health enterprise planning approach and not program specific digital health investments. Standards & Interoperability • The following components were identified as quick wins in terms of what it will take to optimize their maturity: i. Client Registry ii. National Health Data Dictionary (Terminology Service) iii. Shared Health Record/Patient Portal iv. Kenya Health Facility Registry & Master Community Health Unit List • The foundational and critical role of standards was strongly emphasized with guidelines such as FHIR and SMART guidelines highlighted. • Kenya HealthCare Federation (KHF), through their digital health advocacy arm, has self-organized the private sector and set a timeline to consume the Terminology Service—National Health Data Dictionary (NHDD) by December 2023. Services and Applications • Uptake of Point of Care systems such as a national electronic medical record and rollout of eCHIS to 100 000 Community Health Promoters [22]. Infrastructure • Shift in the infrastructure model from a facility to facility-based approach to a digitized Primary Care Network approach. • Adoption of innovative models of digital health infrastructure deployment such as (1) cloud computing and (2) private area networks for connectivity, which reduce long-term costs and increase reliability. • Develop final specifications and requirements for the Kenya Digital Health Superhighway and align it with the overall national Kenya Digital Superhighway. • Formalization of the engagement with the MOICT, Information and Communication Technology Authority (ICTA) and other government agencies like Konza Technopolis to ensure that all components are in place. • Develop a budget and strategy for financing the implementation of all necessary infrastructure. Legislation, Policy and Compliance • The Digital Health Act (2023) was drafted collaboratively—leveraging the Convergence Workshop for public participation and enacted in October 2023 [24]. • Conduction of data protection impact assessments with technical support from the Ministry of Interior and National Administration particularly on matters to do with the establishment of the Client Registry Workforce • Formation of an overarching digital health Kenya Community of Practice consisting of seed sub-communities that featured in the Convergence Workshop with the aim of keeping up the conversations and interactions going and promote knowledge sharing. • The Kenya Health Human Resource Advisory Council is working with the MOH and key stakeholders to (i) better understand the challenges related to the health workforce and building digital health capacity and expertise and (ii) prioritize the role of digital transformation in tracking the health workforce information. • An annual Digital Health Summit to create opportunities for cross learning amongst the stakeholders and the government. • Development of an M&E and Learning plan • Collaborate with the local educational institutions to guide the upskilling of the health workforce through pre-service and in-service knowledge transfer models. Priority Implementation Counties • The launch of a digitized primary care network in Kericho County in October 2023 [24]. • Identification of priority implementation counties by MOH and COG, with the following initial suggestions: i. Nakuru ii. Nandi iii. Kirinyaga

#### Activity prioritization

On the fourth day, all 12 breakout group sessions convened in plenary to share their findings and recommendations. On the fifth day, the DHIPR team and representatives of the Office of the President led a call to action, prioritized high-value activities, received technical and financial commitments from stakeholders and developed an implementation road map highlighting short-, mid- and long-term activities and commitments.

The framework used to identify the priority activities and key outcomes of the workshop as shown in Table 3 is aligned to the WHO/ITU National e-health strategy toolkit—seven strategy building blocks, more commonly referred to as the enabling environment for digital health [25].

### Follow-up

The core strategy team had engaging discussions with the participants, yielding valuable preliminary feedback on how to improve future Convergence Workshops and thereafter documented the output recommendations, priorities and implementation roadmap of the workshop. The priority areas were then communicated to the highest level of the MOH, the Cabinet Secretary, for endorsement.

Picking up on the momentum created from the workshop, the MOH and its stakeholders launched a digitized primary care network in Kapkatet, Kericho County, Kenya, during the national celebrations of Kenya’s Mashujaa (veterans) National day in October 2023, whose theme was UHC. A primary care network is an administrative health region established to efficiently improve access to coordinated primary health care (PHC) services, in line with the Kenya PHC Strategic Framework [25]. A key highlight of the celebrations was the public launch of the Digital Health Act 2023, which had just been enacted a few days before. The Digital Health Act 2023 effectively institutionalized all the key recommendations from the Convergence Workshop, making it possible to lawfully and sustainably implement and realize the envisioned future state of digital health, delivering on the government’s mandate to realize the attainment of UHC through digital health interventions [26, 27].

## LESSONS LEARNT FROM THE KENYAN CONVERGENCE WORKSHOP

The Convergence Workshop employs principles of social network analysis to optimize stakeholder engagement in the digital health sector. By moving away from traditional dynamics where there is a tendency for similar stakeholders to connect and placing emphasis on promotion of inclusivity, the workshop methodology improves how people in the digital health sector connect and share information [28]. This helps spread knowledge and resources more widely across the digital health ecosystem.

Firstly, as a country-led initiative, Convergence Workshops demonstrate a country’s commitment and represent a fundamental step towards establishing digital health sovereignty. The WHO, through its Digital Health Strategy 2020–2025 [29], states that one of its guiding principles is to respect member states’ sovereignty in prioritizing their strategies within their own national and cultural context. By bringing together diverse stakeholders [30], the Convergence Workshop acts as a catalyst for information dissemination and resource sharing, presenting an opportunity to articulate and distil a country’s vision for health system transformation through digital technologies and data, thereby creating buy-in from stakeholders.

Secondly, in the specific case of Kenya, the Convergence Workshop strategically deploys tools such as the WHO Digital Health Atlas and the Global Digital Health Monitor, aiming to (i) streamline digital health interventions, (ii) prevent duplication and (iii) enhance coordination while creating opportunities for new connections, fostering coordination and collaboration.

Thirdly, consensus building in an open, evidence-based session allows all stakeholders to interrogate and align on frameworks for standards and interoperability, further facilitating the co-creation and ownership of a plan for implementation towards realizing the shared vision. Notably, a key learning from the Kenyan Convergence Workshop is that a functional HIE can accelerate innovations and enable differentiated solutions to coexist in a structured framework.

Fourth, breaking down silos within the African digital health landscape is a crucial aspect emphasized by the Convergence Workshop. It ensures that all stakeholders are (i) engaged early, often and consistently and (ii) aligned towards building people-centric interventions based on the identified community needs [31]*.* This contributes to increased efficiency, trust-building and tangible commitments to achieving a shared vision, displaying greater interconnectedness and a more collaborative ecosystem. An outcome of the Convergence Workshop is that the digital health technical working groups formed have regularly scheduled meetings every 3 months.

Fifth, proper documentation of the proceedings and outcomes of a Convergence Workshop allows for the dissemination of information to a wider audience, from which other countries can learn from each other. In addition, it aligns with a key principle for digital development, collaboration, through which the sharing of insights increases efficiency and impact [32].

## CONCLUSION

The Convergence Workshop approach to facilitating the establishment of a HIE allows for a collaborative and evolutionary approach with strong government commitment, promoting ‘interoperability over integration’ [33]. Remarkably, cohesion in thought and commitment from the government and its key stakeholders on the priorities required for Kenya to derive value from its digital health transformation agenda was observed at the workshop. The convergence approach also engages all stakeholders, including end users early, fostering better adoption and acceptance of the CIHMIS [31, 34]. As countries continue to pursue the journey of digital transformation, key considerations should be placed on establishing a solid foundation through consensus to build a robust and sustainable HIE and DPI-H.

## STUDY FUNDING

The funding was provided by the Pan African Health Informatics Association (HELINA) and the German Agency for International Cooperation (GIZ) under agreement number 8129832.

## CONFLICT OF INTEREST

S.W. is the president of HELINA.

## AUTHORS’ CONTRIBUTIONS

Conceptualization—E.W., J.N., R.N., J.W., N.N., S.W., and B.L. Writing (original draft)—E.W. Writing (review and editing)—E.W., N.N., J.N., R.N., J.W., S.W., and B.L. Supervision—S.W. and B.L.- All authors provided comments to and approved this paper.

## DATA AVAILABILITY

No new data were generated or analysed in support of this research.
